# Endothelial Glycocalyx Damage and Arterial Thickness in Patients with Retinal Vein Occlusion (RVO)

**DOI:** 10.3390/jcm14010111

**Published:** 2024-12-28

**Authors:** Emmanouil Korakas, George Pavlidis, Stamatios Lampsas, Chrysa Agapitou, Alexia Risi-Koziona, Aikaterini Kountouri, Loukia Pliouta, Konstantinos Katogiannis, Sotirios Pililis, John Thymis, Evangelos Oikonomou, Gerasimos Siasos, Ignatios Ikonomidis, Vaia Lambadiari, Irini Chatziralli

**Affiliations:** 1 Diabetes Center, 2nd Department of Internal Medicine, Attikon University Hospital, Medical School, National and Kapodistrian University of Athens, 12462 Athens, Greece; mankor-th@hotmail.com (E.K.);; 2 2nd Cardiology Department, Attikon University Hospital, National and Kapodistrian University of Athens, 12462 Athens, Greeceignoik@gmail.com (I.I.); 3 2nd Department of Ophthalmology, Attikon Hospital, National and Kapodistrian University of Athens, 12462 Athens, Greece; lampsas.stam@gmail.com (S.L.); chr.agapitou@gmail.com (C.A.);; 4 3rd Department of Cardiology, Medical School, “Sotiria” Chest Diseases Hospital, National and Kapodistrian University of Athens, 11527 Athens, Greece; 5 Cardiovascular Division, Harvard Medical School, Brigham and Women’s Hospital, Boston, MA 02115, USA

**Keywords:** retinal vein occlusion, arterial stiffness, endothelial glycocalyx, endothelial dysfunction, atherosclerosis

## Abstract

**Background**: Retinal vein occlusion (RVO) is a relatively uncommon condition with a complex pathophysiology. However, its association with traditional cardiovascular risk factors is well established. In this study, we compared arterial stiffness and endothelial function between patients with RVO and healthy controls. **Methods**: We enrolled 28 consecutive patients with RVO, either central (CRVO) or branch (BRVO), and 30 healthy controls. We measured: (i) perfused boundary region of the sublingual arterial microvessels (a marker of endothelial glycocalyx thickness), (ii) pulse wave velocity (PWV), augmentation index (AIx), and central systolic blood pressure (cSBP). **Results**: No statistically significant differences regarding age, gender, and major cardiovascular risk factors were noted between patients and controls. Compared to controls, patients with RVO had higher PBR, PWV, AIx, and cSBP values (*p* < 0.05). For each of these indices, no statistically significant differences were noted between patients with CRVO and BRVO (*p* > 0.05). **Conclusions**: Patients with RVO demonstrated reduced endothelial glycocalyx thickness and increased arterial stiffness compared to healthy controls. These findings further elucidate the role of atherosclerosis and endothelial dysfunction in the pathophysiology of the disease and indicate the need for the evaluation of subclinical cardiovascular disease in such patients.

## 1. Introduction

Following diabetic retinopathy, retinal vein occlusion (RVO) is the second most common retinal vasculopathy which, if left untreated, can lead to serious clinical implications such as visual impairment and, eventually, blindness [1]. Its classification is based on the location of the lesion, and it includes central (CRVO) and branch (BRVO) retinal vein occlusion. Although its pathogenesis is still to be fully elucidated, multiple pathophysiological pathways have been proposed [2]. Traditional cardiovascular risk factors, such as arterial hypertension, and diabetes mellitus have a well-established association with the development of RVO and, in a vicious cycle, RVO per se is a significant risk factor for cardiovascular disease (CVD) [3,4]. However, in recent years, with the development of advanced imaging techniques, it has been shown that markers of subclinical atherosclerotic disease, like arterial stiffness and endothelial dysfunction, are also associated both with the pathogenesis of RVO and, even more importantly, with the risk of developing the disease, especially in populations of high cardiometabolic burden [5,6].

Increased arterial stiffness has an independent causal and prognostic association with cardiovascular morbidity and mortality [7]. Patients with diabetes mellitus (DM) or arterial hypertension have impaired elastic properties of the large vessels, and such abnormalities can also be observed in any setting of insulin resistance, even in the absence of overt diabetes [8]. On the other hand, endothelial dysfunction can be depicted through the integrity of endothelial glycocalyx, which is a mesh of glycoproteins, proteoglycans, and associated glycosaminoglycans, covering the endothelium and playing a vital role in permeability, mechanotransduction, and in other functions like immunity and hemostasis [9]. The high levels of intercellular adhesion molecule-1 (ICAM-1) in the retinal endothelial cells is a major contributing factor to the development of RVO; on the other hand, an-ti-VEGF (vascular endothelial growth factor) treatments are the pillars of RVO management [10]. Therefore, the role of intact endothelial integrity, along with the prevention of functional changes which could promote a pro-thrombotic environment, are key factors in the prevention of RVO. Reduced glycocalyx thickness, which is translated into increased endothelial permeability, can be found across the whole spectrum of cardiometabolic disease, and recent data have shown that is an independent risk factor for vascular complications in any condition entailing chronic, low-grade inflammation [11,12]. Although some data regarding the association between RVO and arterial stiffness can be found in recent reports, there are no previous study findings regarding endothelial glycocalyx thickness and RVO in the existing literature. In this study, we aimed to determine the association of RVO incidence with arterial stiffness and endothelial glycocalyx thickness in patients with no previous history of overt cardiovascular disease.

## 2. Materials and Methods

### 2.1. Study Design

In this prospective, case-control study, we included 28 consecutive naïve and previously untreated patients with RVO (either CRVO or BRVO) and 30 consecutive age- and gender-matched healthy controls, between 1 September 2023 and 30 May 2024. Patients with RVO were recruited from the 2nd Department of Ophthalmology, Attikon University Hospital, and the controls were recruited from the Cardiometabolic outpatient clinic of Attikon Hospital. All patients were between 18 and 80 years of age. The diagnosis of RVO was based on clinical criteria (retinal hemorrhages, dilated and tortuous veins, and flame-shaped or dot-blot hemorrhages) and it was confirmed with imaging techniques. None of the subjects had a history of CVD, active malignancy, chronic kidney disease, systemic or other inflammatory diseases, or ocular diseases, or any history of ocular surgery. None of the female subjects was pregnant or received hormonal replacement.

### 2.2. Clinical Measurements

All participants underwent best-corrected visual acuity (BCVA) measurement using Snellen charts slit-lamp examination, dilated fundoscopy, SD-OCT, and fundus fluorescein angiography (FFA) (Spectralis HRA+OCT, Heidelberg Engineering, Heidelberg, Germany).

Moreover, arterial stiffness was measured through the carotid-femoral Pulse Wave Velocity (PWV), augmentation index (AIx), and central aortic pressures [central systolic (cSBP) and diastolic blood pressure (cDBP)] using tonometry by Complior (Alam Medical, Vincennes, France). Normal values were PWV < 10 m/s. Additionally, the perfused boundary region (PBR) of the sublingual arterial microvessels (5–25 μm in diameter) was measured using Sidestream Dark Field imaging (Microscan, Glycocheck, Microvascular Health Solutions Inc, Salt Lake City, UT, USA). The PBR is an area that results from the separation between the column of the red blood cells and plasma on the luminal vascular surface. The more increased the PBR value, the more reduced glycocalyx thickness is. The evaluation of endothelial dysfunction through the calculation of PBR holds significant clinical value in the management of microvascular diseases [13,14]. Damage or thinning of the glycocalyx, often reflected in elevated PBR values, is an early marker of endothelial dysfunction [15,16,17]. Impaired endothelial glycocalyx function has been associated with subclinical microvascular injury, cardiovascular disease progression, and can also have a predictive value in several interventions [18,19,20]. The study was conducted according to the Declaration of Helsinki. The study was approved by the Institutional Review Board of Attikon University Hospital (reference number 519/2023, approval date 3 August 2023). Written informed consent was obtained prior to the patients’ participation.

### 2.3. Statistical Analysis

Statistical analysis was conducted using the Statistical Package for Social Sciences (IBM SPSS Statistics for Windows, Version 26.0. Armonk, NY, USA). Continuous variables were expressed as mean ± standard deviation. Differences in continuous variables were evaluated using one-way analysis of variance (ANOVA). Categorical variables were presented as numbers with corresponding percentages and were analyzed by performing the chi-squared test or Fisher’s exact test. A *p*-value < 0.05 was considered statistically significant.

## 3. Results

### 3.1. Baseline Characteristics of the Study Population

In this analysis, we included 28 patients, 12 of whom with CRVO and 16 with BRVO, and 30 consecutive healthy controls. The mean age was 69 ± 9, 71 ± 5, and 67 ± 11 years, respectively, among the three groups. No major differences were reported between the compared groups in age, male–gender ratio, and in the prevalence of major cardiovascular risk factors (arterial hypertension, diabetes mellitus, dyslipidemia, and smoking) (Table 1).

### 3.2. Arterial Wall Properties and RVO

**Figure 1:**
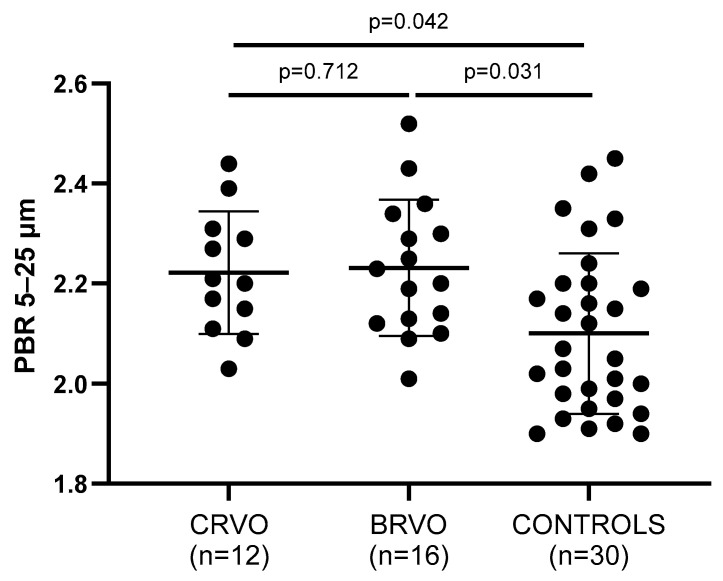
Comparison of PBR values between patients and controls. Both CRVO and BRVO patients had significantly higher PBR values than controls, but no statistically significant differences were noted between CRVO and BRVO patients. CRVO: central retinal vein occlusion; BRVO: branch retinal vein occlusion; PBR: perfused boundary region.

Endothelial dysfunction—being assessed by the PBR of the sublingual arterial microvessels—was significantly impaired in both CRVO and BRVO patients when compared to healthy controls, since higher PBR values were reported in RVO subjects (Figure 1). In particular, not only PBR 5–25 (*p* = 0.042, *p* = 0.031), but also PBR 5–9 (*p* = 0.045, *p* = 0.001), PBR 10–19 (*p* = 0.006, *p* = 0.048), and PBR 20–25 (*p* = 0.007, *p* = 0.001) reported impaired endothelial function when CRVO and ΒRVO patients were compared with healthy controls, respectively.

**Figure 2:**
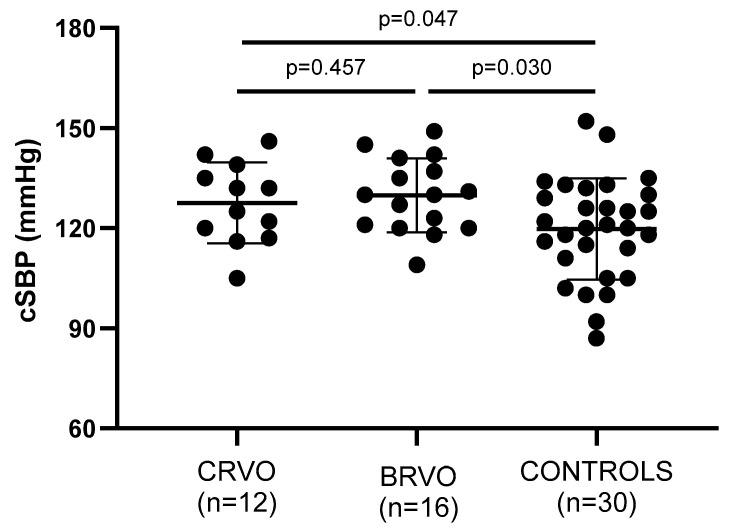
Comparison of cSBP values between patients and controls. Both CRVO and BRVO patients had significantly higher PBR values than controls, but no statistically significant differences were noted between CRVO and BRVO patients. CRVO: central retinal vein occlusion; BRVO: branch retinal vein occlusion; cSBP: central systolic blood pressure.

**Figure 3:**
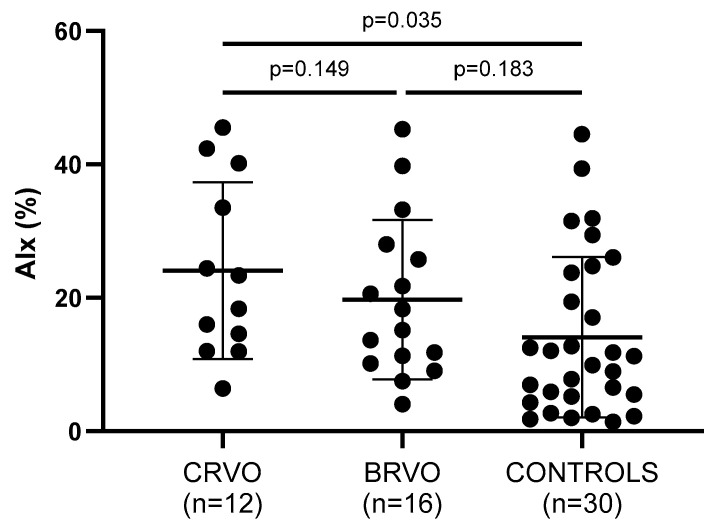
Comparison of AIx values between patients and controls. CRVO, but not BRVO, patients had significantly higher AIx values than controls. No statistically significant differences were noted between CRVO and BRVO patients. CRVO: central retinal vein occlusion; BRVO: branch retinal vein occlusion; AIx: augmentation index.

**Figure 4:**
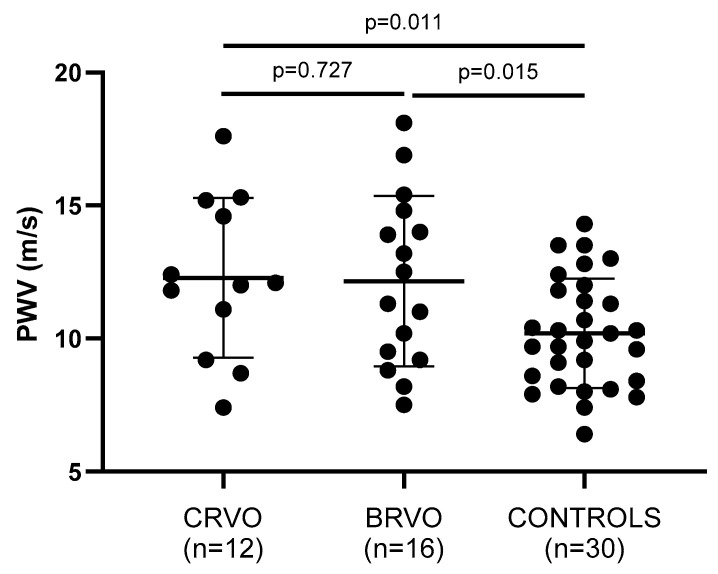
Comparison of PWV values between patients and controls. Both CRVO and BRVO patients had significantly higher PWV values than controls. No statistically significant differences were noted between CRVO and BRVO patients. CRVO: central retinal vein occlusion; BRVO: branch retinal vein occlusion; PWV: pulse wave velocity.

Arterial wall stiffness—being assessed by PWV, AIx, and cSBP—was significantly increased in both CRVO and ΒRVO patients when compared to healthy controls (Table 2). Particularly, PWV (*p* = 0.011, *p* = 0.015), AIx (*p* = 0.035, *p* = 0.183), and cSBP (*p* = 0.047, *p* = 0.030) showed to be increased in the compared groups, respectively (Figure 2, Figure 3 and Figure 4).

**Figure 5:**
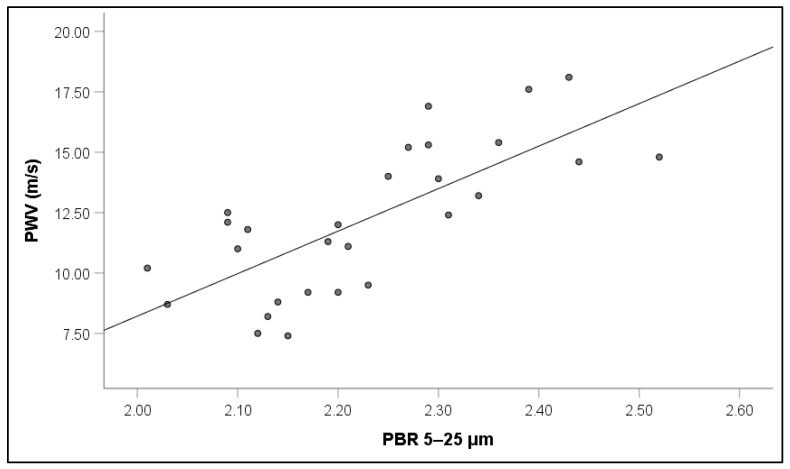
Correlation between pulse wave velocity (PWV) and perfused boundary region (PBR) of the sublingual microvessels with diameter between 5 and 25 μm in patients with retinal vein occlusion.

**Figure 6:**
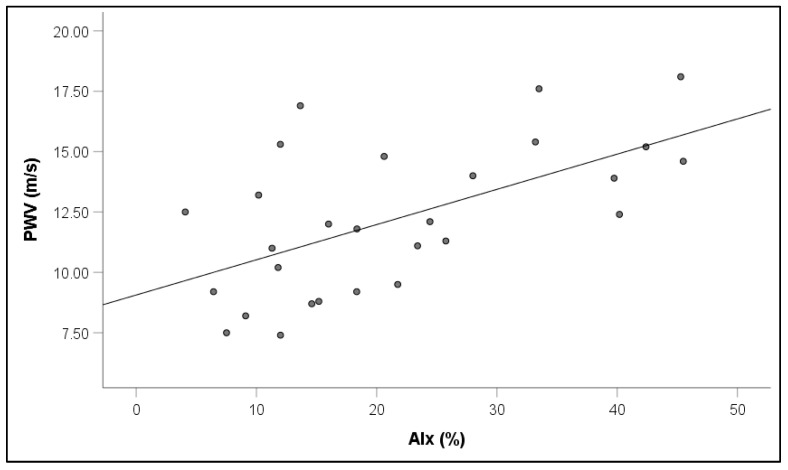
Correlation between pulse wave velocity (PWV) and augmentation index (AIx) in patients with retinal vein occlusion.

Notably, increased PWV values in RVO were associated with increased PBR5–25 (r = 0.65, *p* = 0.001) (Figure 5), PBR20–25 (r = 0.60, *p* = 0.003), cSBP (r = 0.64, *p* = 0.001), and AIx (r = 0.31, *p* = 0.042) (Figure 6).

## 4. Discussion

In this study, we showed that markers of arterial stiffness, namely PWV, AIx, and cSBP, were increased in patients with RVO compared to healthy controls. In addition, patients with RVO had decreased endothelial glycocalyx thickness compared to healthy subjects, which is indicative of impaired endothelial function. These results were independent of the type of RVO (CRVO or BRVO) and the presence of traditional cardiovascular risk factors.

The pathophysiology of RVO is complicated and has not yet been fully explained. However, it is clear that structural changes in collagen due to aging contribute to atherosclerosis of the retinal arteries, leading to the remodeling of the vascular wall [21]. As retinal arteries and veins share a common adventitial sheath at arteriovenous crossings, increased rigidity of the arterial wall can lead to vein compression and, potentially, occlusion. In cases of established atheromatosis, like in DM and arterial hypertension, this risk is augmented [22]; however, the elastic properties of the vascular wall may play an even more crucial role in RVO pathogenesis compared to its diameter or other structural characteristics. PWV is a well-established, non-invasive method of evaluating arterial stiffness [23]. In our study, we showed that PWV was significantly increased in patients with RVO, regardless of its subtype. These results are in accordance with most previous reports, though several remarkable differences need to be noted. In the study by Gouliopoulos et al. [6], with a sample size similar to ours, PWV was significantly higher in patients compared to controls, albeit slightly lower numerically compared to our cohort (11.37 m/s vs 12.2 m/s). However, contrary to our study, the subtype of RVO was not reported. In addition, no association between PWV and blood pressure was noted in patients with RVO, which implies that structural changes and not blood pressure are the main pathogenetic mechanism that exacerbates arterial stiffness in patients with RVO. In our cohort, cSBP, which is considered a more reliable predictive factor for cardiovascular events than peripheral blood pressure, was also measured, and it was increased in RVO patients compared to controls. Increased PWV values were also shown in the study by Nakazato et al. [24]; however, the sample included only patients with BRVO, as it was the case in the study by Chen et al. [25]. Similar results were reported by Karadag et al. [26] and Kaderli et al. [5], but the sample included only patients with CRVO, which is a significant methodological difference compared to our study. Furthermore, it must be noted that in the last study, body mass index (BMI) was increased in patients with BRVO compared to controls and, therefore, the dysmetabolic effect of obesity on the results cannot be ruled out. In the same notion, in the study by Bozkurt et al. [27], patients and controls had significant baseline differences regarding medical history of diabetes, arterial hypertension, and dyslipidemia and, therefore, the role of increased PWV in the incidence of RVO should be interpreted with caution. On the contrary, in our cohort, subjects were matched for cardiovascular risk factors, similar to the study by Lyu et al. [28]. On the other hand, AIx has been reported only once in the literature, and it was increased in CRVO but not BRVO patients, as in our cohort.

Endothelial dysfunction has long been recognized as a pivotal risk factor for cardiometabolic disease [29]. As far as ocular disease is concerned, its role in the pathogenesis of glaucoma and diabetic retinopathy is well established [30,31]. The decreased bioavailability of nitric oxide (NO) is at the core of this pathophysiologic process, leading to impaired elasticity and hypercoagulability, a state which often precedes overt atherosclerotic lesions. However, data regarding the role of endothelial dysfunction specifically in the natural course of RVO are still scarce. In our study, we showed that PBR was increased in patients with RVO, a fact that implies a direct association between impaired endothelial glycocalyx thickness and the development of the disease. To our knowledge, this is the first study to use endothelial glycocalyx thickness as a marker of endothelial function in RVO. In general, endothelial function is deteriorated in this population, but it is assessed via flow-mediated dilation (FMD). In the study by Gouliopoulos et al. [6], FMD was reduced in patients compared to controls, independently of the presence of cardiovascular risk factors, contrary to the cohort of Bozkurt et al. [27]. Tanano et al. showed that FMD was seriously reduced in patients with BRVO, but CRVO patients were excluded from their sample, which is a notable difference compared to our cohort [32]. However, a remarkable finding was that FMD values were similar to patients with T2DM. In addition, the PBR values in patients with RVO in our study are comparable numerically to the PBR values in patients with T2DM or arterial hypertension in other reports [33,34]. Such observations confirm the common pathogenetic pathways in retinal vein occlusion and cardiometabolic disease and explain the association between RVO and cardiovascular risk factors with mechanisms beyond atherosclerosis.

Our study has limitations. The sample size is relatively small, although it is comparable to other reports which also have a limited sample size due to the low incidence of the disease. Furthermore, it is an observational study in a single center, which does not allow for robust causal relationships to be established. However, subjects were matched for age, gender, and cardiovascular risk factors, which renders our results independent of any confounding factors. Finally, measuring endothelial markers, such as the perfused boundary region (PBR) or biomarkers like circulating endothelial progenitor cells and glycocalyx integrity markers, may provide valuable insight into the patient’s vascular health. However, these markers may not be cost-effective or clinically necessary for routine use. Prioritizing stratification in patients with unexplained or recurrent RVO, young patients without traditional risk factors, or those with additional signs of systemic vascular disease might be more appropriate.

## 5. Conclusions

In our study, we showed that patients with RVO have decreased endothelial glycocalyx thickness and increased arterial stiffness compared to healthy controls. These findings indicate the role of atherosclerosis and endothelial dysfunction in the pathophysiology of the disease. Of course, such techniques are not readily and widely available in the healthcare infrastructure, so the application of these markers in everyday clinical practice cannot yet be a viable preventive measure. However, the evaluation of such markers of subclinical cardiovascular disease can contribute to cardiovascular risk stratification and the individualized treatment of patients with retinal vein occlusion and, thus, more extensive research is warranted to extrapolate these results to a wider population.

## Figures and Tables

**Table 1:** Clinical and demographic characteristics of the study population.

	CRVO (n = 12)	ΒRVO (n = 16)	Controls (n = 30)	*p*-Value
Age (years)	69 ± 9	71 ± 5	67 ± 11	0.583
Male gender (n, %)	6 (50)	8 (50)	14 (47)	0.967
	Cardiovascular risk factors (n, %)			
Arterial hypertension	8 (67)	11 (69)	20 (67)	0.915
Diabetes mellitus	5 (42)	7 (44)	11 (37)	0.798
Dyslipidemia	7 (58)	8 (50)	15 (50)	0.748
Smoking	5 (42)	8 (50)	13 (43)	0.517

Data are presented as mean ± standard deviation, or number (%). Continuous variables were compared with one-way ANOVA. Binary variables were compared with the chi-squared test. CRVO: central retinal vein occlusion; BRVO: branch retinal vein occlusion.

**Table 2:** Arterial stiffness and endothelial function markers in study population.

	CRVO (n = 12)	ΒRVO (n = 16)	Controls (n = 30)	*p*-Value
PBR 5–25 μm	2.22 ± 0.13 *	2.23 ± 0.14 **	2.10 ± 0.16	0.024
PBR 5–9 μm	1.29 ± 0.14 *	1.35 ± 0.15 ††	1.21 ± 0.11	0.004
PBR 10–19 μm	2.39 ± 0.14 †	2.36 ± 0.26 **	2.21 ± 0.25	0.045
PBR 20–25 μm	2.97 ± 0.49 †	2.95 ± 0.25 ††	2.55 ± 0.39	0.002
cSBP (mmHg)	127 ± 12 *	129 ± 11 **	119 ± 16	0.033
cDBP (mmHg)	76 ± 7	77 ± 6	76 ± 8	0.897
AIx (%)	24.1 ± 13.6 *	19.7 ± 12.0	14.0 ± 12.1	0.007
PWV (m/s)	12.3 ± 3.1 *	12.2 ± 3.2 **	10.1 ± 2.0	0.009

Data are presented as mean ± standard deviation. The variables were compared with one-way ANOVA and the *p*-values are presented. * *p* < 0.05, † *p* < 0.01 obtained by post hoc analysis between CRVO and controls. ** *p* < 0.05, †† *p* < 0.01 obtained by post hoc analysis between BRVO and controls. Significant differences at *p* < 0.05 level were not observed for comparisons of CRVO and BRVO. CRVO: central retinal vein occlusion; BRVO: branch retinal vein occlusion; PBR: perfused boundary region; cSBP: central systolic blood pressure; AIx: augmentation index; PWV: pulse wave velocity.

## Data Availability

Dataset on request.
